# Inner retinal layer thickness reflects plasma biomarkers in preclinical Alzheimer’s disease

**DOI:** 10.3389/fmed.2026.1800287

**Published:** 2026-05-18

**Authors:** Jane W. Chan, Ziyuan Wang, Emily Xu, Ibrahim Abboud, Aya Alhasany, Sophia Xu, Xiaomeng Wu, Natalie Astraea, Fei Jiang, Zhihong Jewel Hu

**Affiliations:** 1 Department of Ophthalmology, David Geffen School of Medicine at UCLA, Los Angeles, CA, United States; 2 Doheny Image Analysis Laboratory, Doheny Eye Institute, Pasadena, CA, United States; 3 Analytical Biochemistry Core Laboratory, Huntington Medical Research Institute, Pasadena, CA, United States; 4 Department of Epidemiology and Biostatistics, University of California, San Francisco, San Francisco, CA, United States

**Keywords:** Alzheimer’s disease, deep-learning segmentation algorithm, plasma glial fibrillary acidic protein, plasma neurofilament-light chain, plasma phosphorylated-tau217, spectral-domain optical coherence tomography

## Abstract

**Purpose/background:**

Few studies have examined layer- and region-specific retinal thickness in relation to plasma Alzheimer’s disease (AD) biomarkers in cognitively normal adults at increased risk for AD. We conducted an exploratory pilot study to examine whether thickness measurements of the retinal nerve fiber layer (RNFL), ganglion cell layer (GCL), inner plexiform layer (IPL), inner nuclear layer (INL), and outer plexiform layer (OPL) were associated with plasma AD biomarkers.

**Methods:**

Spectral-domain optical coherence tomography was performed on 20 eyes from 11 cognitively normal participants enriched for AD risk using a low plasma Aβ42/40 ratio (<0.10). Retinal layers were segmented using a novel deep-learning algorithm, followed by manual review and refinement by trained graders when needed. Associations between retinal thickness and plasma neurofilament-light chain (NfL), glial fibrillary acidic protein (GFAP), Aβ42/40, p-tau217, and p-tau181 were evaluated using ridge regression with participant-level bootstrap resampling.

**Results:**

Exploratory relationships suggest that thinner INL and GCL measurements are associated with higher p-tau217 and GFAP levels.

**Conclusion:**

Larger, longitudinal studies with confirmatory AD biomarker characterization are needed to validate these preliminary findings to determine how retinal OCT adds complementary value to plasma biomarkers for early AD risk assessment.

## 1 Introduction

Alzheimer’s disease (AD) is a progressive neurodegenerative disorder that evolves over many years before clinical symptoms become apparent and these early changes are detectable in plasma and CSF biomarkers (1–3). Early identification of individuals at risk for AD is critical for advancing preventive strategies and for selecting participants in disease-modifying clinical trials.

The retina is an accessible extension of the central nervous system that shares embryologic, vascular, and neuronal features with the brain (4). Retinal imaging modalities have been investigated for the early detection of dementia and Alzheimer’s disease (AD). Retinal vascular metrics derived from fundus photography have been associated with dementia-related differences in vessel caliber, tortuosity, fractal dimension, and retinopathy, supporting the concept that retinal microvascular imaging may reflect cerebral microvascular pathology (5). Optical coherence tomography angiography (OCTA) has expanded this vascular approach by showing reduced superficial retinal vessel density and enlargement of the foveal avascular zone in AD, although between-study heterogeneity remains substantial (6, 7). In parallel, hyperspectral retinal imaging has been explored as a noninvasive strategy for detecting beta-amyloid and p-tau proteins (8). Within this context, spectral-domain optical coherence tomography (SD-OCT) adds complementary evidence by providing noninvasive, technically simple, inexpensive, and layer-specific retinal measurements (9, 10). Meta-analytic and recent systematic-review data indicate that SD-OCT can detect thinning of the ganglion cell-inner plexiform layer, ganglion cell complex, macular thickness/volume, and peripapillary retinal nerve fiber layer in AD (9, 10). Biomarker-defined cohorts at the preclinical AD stage suggest that analogous structural changes may appear before cognitive impairment (11–13). Because these findings have been inconsistent, there is an unmet clinical need for longitudinal standardization (14).

Current plasma biomarkers, including Aβ42/40, p-tau217, p-tau181, neurofilament-light chain (NfL), and glial fibrillary acidic protein (GFAP), are increasingly used as minimally invasive correlates of amyloidosis, tau pathology, neuroinflammation, and neurodegeneration (1, 2, 15). Although plasma biomarkers and neuroimaging are valuable for risk assessment and disease monitoring, they may not fully capture the spatial heterogeneity and temporal evolution of early AD-related changes that retinal imaging might offer. For example, SS-OCTA showed the spatial progression of the disease, starting from the retinal periphery toward the macula. The choriocapillaris flow deficit in the outer macular ring was significantly more predictive of CSF Aβ_42_/tau compared to the inner ring. These results are an example highlighting how SS-OCTA metrics can complement biofluid markers to define the transitional stages from normal aging to the preclinical AD stage (16).

Optical coherence tomography (OCT) provides high-resolution, non-invasive, three-dimensional assessment of retinal architecture (4, 9). Lopez-de-Eguileta et al. (13) reported thinning of the ganglion cell layer (GCL) in cognitively normal adults at increased risk for AD. Other studies further showed these changes in patients with mild cognitive impairment (MCI) and AD (17–20). However, the peri- and parafoveal inner retinal layers have not yet been extensively studied in relation to plasma AD biomarkers.

In this cross-sectional pilot study, we investigated whether layer-specific inner retinal thickness, measured using a novel deep-learning-based segmentation algorithm, correlates with established plasma biomarkers of AD-related pathology and neurodegeneration in cognitively normal older adults at increased risk for AD. Because this was an exploratory pilot study, our study aim was to identify the size effect estimates, variance, and directional patterns as continuous variables in our preclinical AD cohort. Our preliminary results could then be validated in future larger preclinical AD cohorts compared to normal age-matched controls.

## 2 Methods

### 2.1 Study participants

Eleven participants, recruited from a large brain aging cohort at the Huntington Medical Research Institute, Pasadena, CA, had a pathological Aβ42/40 ratio based on the plasma cut-off ratio of Aβ42/Aβ40 < 0.10 (mean ± SD = 0.05 ± 0.01), as determined by Fonteh et al. (20) which distinguishes cognitively normal individuals with a lower risk (normal aging) from those at a higher risk of cognitive decline (preclinical AD).

CSF Abeta 42/total tau was used to initially classify the pre-clinical AD cohort, according to the methods section described in Harrington et al. (21). Cognitively healthy-pathological CSF Abeta 42/tau (CH-PAT) were distinguished from cognitively healthy-normal CSF Abeta 42/tau (CH-NAT). The authors of this study showed that the abnormal CSF Abeta42/40 cut-off point predicted conversion of CH-NAT to CH-PAT within a four-year period. Our current pilot study cohort was recruited from this initial CH-PAT group.

Although the plasma Abeta42/40 cut-off is considered an evolving screening biomarker, our cut-off value was consistent with the CSF Abeta42/tau cut-off in classifying this CH-PAT group, which we labeled as pre-clinical AD. We defined pre-clinical AD as cognitively normal adults at increased risk for Alzheimer’s disease. Participants were enriched for AD risk using this abnormal plasma Aβ42/40 ratio as an operational study cutoff rather than a definitive diagnostic threshold for preclinical AD.

Inclusion criteria were cognitively normal adults who had undergone neurological and neuropsychological assessment, met study criteria for normal cognition, and had gradable macular SD-OCT scans with signal strength >30. Normal cognition was defined by the absence of symptoms, a Clinical Dementia Rating (CDR) score of 0, a Functional Activity Questionnaire (FAQ) total score of 0, neuropsychological performance within at least 1 standard deviation of age- and education-adjusted norms, and no diagnosis of mild cognitive impairment (MCI) (21, 22). Exclusion criteria included ocular disease, neurological disease, systemic disease likely to affect retinal structure, and ungradable OCT scans.

A total of 22 possible eyes from 11 participants were screened, and 20 eyes were analyzed after exclusion of 2 eyes because of inadequate image quality. Blood collection and OCT imaging were performed within a 6-month period.

The study was approved by the Institutional Review Board of the University of California, Los Angeles (IRB #21-001404). Written informed consent was obtained from all participants.

### 2.2 Retinal scanning methods

Spectral-domain OCT macular volumes (20 × 20 degrees, 97 B-scans/lines; Spectralis SD-OCT, Heidelberg) centered on the fovea were acquired for all participants at the Doheny Eye Center/University of California, Los Angeles (UCLA). All OCT images with signal strength >30 from the Heidelberg Spectralis platform were exported and transferred to the Doheny Image Analysis Laboratory (DIAL). In this study, we segmented the five retinal layers/spaces relevant to the analysis: the retinal nerve fiber layer (RNFL), ganglion cell layer (GCL), inner plexiform layer (IPL), inner nuclear layer (INL), and outer plexiform layer (OPL).

Manual delineation of these layers in volumetric OCT images is extremely time-consuming and labor-intensive. To address this challenge, we used a deep-learning-derived, graph-based algorithm to automatically identify the key retinal boundaries associated with these layers (23–26). Our segmentation approach used a shortest-path algorithm enhanced with probability maps generated through a fully convolutional neural network to delineate the relevant boundaries in SD-OCT images. Following automated segmentation, four trained DIAL graders reviewed every B-scan and manually refined boundary positions when necessary, using 3D OCTOR to ensure accurate layer thickness measurements. After verification, 3D OCTOR calculated retinal layer thicknesses and generated Early Treatment Diabetic Retinopathy Study (ETDRS) subfield thickness maps.

### 2.3 Biochemistry methods

Blood samples were collected in ethylenediaminetetraacetic acid (EDTA) tubes at the Huntington Medical Research Institutes (HMRI) in Pasadena, California, in accordance with IRB-approved procedures. Blood samples were centrifuged at 3,000 RCF and 20 °C for 5 min to separate the supernatant from the cell pellet. The plasma samples were carefully aliquoted, deidentified, barcoded, and stored at −80 °C. On the testing date, aliquots were retrieved from cold storage, thawed on ice at the benchtop, and vortexed to ensure homogeneity before assay performance. Plasma biomarkers were characterized by electrochemiluminescence using the MSD SQ120MM platform and the MSD V-PLEX Plus Aβ Peptide Panel 1, S-PLEX Neurology Panel, S-PLEX p-tau217, and S-PLEX p-tau181 kits (Meso Scale Discovery, Rockville, Maryland), adhering to manufacturer specifications and approved laboratory and biobanking procedures.

### 2.4 Statistical analysis

In this pilot study, we used ridge regression with bootstrap resampling to examine the associations between the retinal layer thicknesses as predictors and the plasma biomarkers as outcomes. Accounting for potential correlations between eyes, bootstrap resampling (*N* = 100) was used at the patient level to calculate the 95% confidence intervals (CIs) for each predictor effect. Since the aim of this study was to obtain size effect estimates and variance to calculate the study power for a future larger longitudinal clinical trial, the inclusion of both eyes was necessary to improve statistical power in this small sample size. Bootstrap resampling at the patient level was performed to preserve subject-level variability while accounting for inter-eye correlation so that statistical assumptions would not be violated. All models were adjusted for age and normalized for skewed distributions of biomarker values by natural log transformation. The other covariates, such as sex, vascular risk factors, and axial length were essentially homogenous in our small cohort, which consisted of mostly females with minimal to no vascular risk factors. The refractive error for all participants was <4.00 D. We reported the size effect estimates and the 95% Cis. Statistical significance was defined as the predictors with 95% CIs that did not cross zero, not by the conventional *p*-values of < 0.05. For a preliminary ridge analysis, it is more reasonable to view the results as exploratory. A Bonferroni correction would not likely rescue invalid bootstrapped p-values in our penalized regression analysis. Therefore, a multiple comparisons test was not applied in this setting (27).

## 3 Results

### 3.1 Study cohort

Our pilot study included 20 eyes from 11 cognitively normal participants at increased risk for AD, as indicated by low plasma Aβ42/40 (mean ± SD: 0.05 ± 0.01). The mean age was 74.9 ± 10.4 years, and 9 of 11 participants (82%) were female. Demographic and clinical characteristics are summarized in Table 1.

**Table 1:** Demographic and clinical features of the study cohort.

Characteristic	Value
Total number of subjects	*N* = 11
Total number of eyes (analytic)	*N* = 20
Age	Mean = 74.9; SD = 10.4 years
Female participants	*N* = 9; 82%
ApoE ε3/3	*N* = 9; 82%
ApoE ε3/4	*N* = 2; 18%
Plasma Aβ42/40 ratio (risk-enrichment cutoff < 0.10)	*N* = 11; mean = 0.05; SD = 0.01
Mini-Mental State Examination (MMSE) score	*N* = 11; mean = 27.5; SD = 0.7
Best-corrected visual acuity	*N* = 11; mean = 20/25; SD = 1.2

Normal cognition was defined by the full neurological and neuropsychological inclusion criteria, as described in the methods, not by MMSE alone.

### 3.2 Relationships between inner retinal layers and plasma AD biomarkers

Exploratory ridge regression analyses identified directional relationships between inner retinal layer thickness and plasma biomarkers (Table 2). Because this was a small pilot study without multiple-comparison correction, statistically non-significant findings are described as trends.

**Table 2:** Ridge regression results ranked by absolute effect size who 95% confidence intervals did not cross zero.

Predictor	Outcome	Estimate	95% Lower CI	95% Upper CI	Direction	CI width	*p*-value
IPL outer nasal	NfL	−744.4	−2940.4	−105.3	Negative	2835.1	0.2
IPL outer temporal	NfL	−346.3	−1126.8	−24.5	Negative	1102.3	0.2
INL superior sum	GFAP	−266.9	−575.1	−28.1	Negative	547.0	0.1
IPL outer nasal	GFAP	−231.6	−606.7	−22.7	Negative	584.0	0.1
RNFL inner inferior	GFAP	−192.4	−372.9	−45.1	Negative	327.8	0.01
IPL outer temporal	GFAP	−170.5	−543.8	−45.0	Negative	498.8	0.14
INL inner temporal	GFAP	−164.4	−266.6	−46.7	Negative	219.9	0.0
OPL outer inferior	GFAP	−148.6	−328.0	−16.6	Negative	311.4	0.05
OPL inner nasal	GFAP	108.6	19.4	221.8	Positive	202.4	0.03
GCL superior sum	GFAP	−105.4	−233.7	−1.1	Negative	232.6	0.07
GCL inner temporal	GFAP	−92.5	−192.7	−25.7	Negative	167.0	0.0
GCL inner nasal	GFAP	−90.2	−186.6	−17.7	Negative	168.9	0.01
INL superior sum	ptau217	−24.8	−51.6	−5.6	Negative	46.0	0.04
IPL outer nasal	ptau217	−17.0	−35.2	−0.7	Negative	34.5	0.02
INL inner temporal	ptau217	−12.5	−26.1	−1.4	Negative	24.7	0.05
IPL outer temporal	ptau217	−11.5	−26.8	−0.1	Negative	26.7	0.1
GCL superior sum	ptau217	−8.8	−18.7	−0.5	Negative	18.2	0.03
GCL inner temporal	ptau217	−6.6	−13.6	−0.8	Negative	12.7	0.01
GCL inner nasal	ptau217	−4.9	−9.7	−0.1	Negative	9.6	0.02
OPL outer nasal	Abeta42/40	0.1	0.1	0.1	Positive	0.0	0.04
OPL outer inferior	Abeta42/40	0.1	0.1	0.1	Positive	0.0	0.0
OPL outer temporal	Abeta42/40	0.1	0.1	0.1	Positive	0.0	0.0
OPL temporal sum	Abeta42/40	0.1	0.1	0.1	Positive	0.0	0.0
OPL superior sum	Abeta42/40	0.1	0.1	0.1	Positive	0.0	0.0
GCL inner nasal	Abeta42/40	0.0	0.0	0.0	Positive	0.0	0.1
GCL inner temporal	Abeta42/40	0.0	0.0	0.0	Positive	0.0	0.02
GCL superior sum	Abeta42/40	0.0	0.0	0.0	Positive	0.0	0.1
IPL outer nasal	Abeta42/40	0.0	0.0	0.0	Positive	0.0	0.01
IPL outer temporal	Abeta42/40	0.0	0.0	0.0	Positive	0.0	0.06
INL inner temporal	Abeta42/40	0.0	0.0	0.0	Positive	0.0	0.1
INL superior sum	Abeta42/40	0.0	0.0	0.0	Positive	0.0	0.4

Confidence intervals and *p*-values are descriptive. RNFL, retinal nerve fiber layer; GCL, ganglion cell layer; IPL, inner plexiform layer; INL, inner nuclear layer; OPL, outer plexiform layer; superior sum, superior summated regions including the superior outer and superior inner regions; CI, confidence interval; Abeta42/40, amyloid-beta 42/40; NfL, neurofilament-light chain; GFAP, glial fibrillary acidic protein; p-tau217, phosphorylated tau 217; p-tau181, phosphorylated tau 181.

Among the retinal regions evaluated, the outer nasal IPL showed the largest-magnitude negative coefficient for plasma NfL (*β* = −744.4; 95% CI, −2940.4 to −105.3), and the outer temporal IPL also showed a negative coefficient (*β* = −346.3; 95% CI, −1126.8 to −24.5). However, these bootstrap-based results did not reach statistical significance (both *p* = 0.20) and are interpreted as directional trends.

Multiple retinal layers showed negative associations with plasma GFAP, most notably the INL inner temporal, GCL inner temporal, RNFL inner inferior, and GCL inner nasal regions. Additional negative trends were observed in the INL superior sum (summated regions of outer and inner superior sectors), IPL outer nasal and outer temporal, OPL outer inferior, and GCL superior sum. Only the OPL inner nasal showed a positive association. Overall, these exploratory findings suggest that thinner inner retinal layers, particularly the INL and GCL, may be associated with higher plasma GFAP levels, with regional variability in the OPL.

Thinner INL, IPL, and GCL regions were further associated with higher plasma p-tau217. The clearest negative associations were observed in the following locations: (1) INL superior sum, (2) INL inner temporal, (3) IPL outer nasal, and (4) GCL inner nasal, inner temporal, and superior sum. Associations with p-tau181 were weaker and non-significant. These patterns support further study of layer-specific retinal thinning as a correlate of tau-related change.

OPL thickness showed positive associations with plasma Aβ42/40 across all OPL regions. In contrast, associations in the GCL, IPL, and INL were small or absent.

## 4 Discussion

Our exploratory findings suggest that higher plasma GFAP levels may be associated with parafoveal thinning in the RNFL, GCL, and INL together with regional thickening in the OPL. This pattern is biologically plausible. Histomorphometric studies have shown increased GFAP reactivity and astrocytic activation in the foveal/parafoveal regions of AD retinas (28), and clinical work has linked elevated plasma GFAP levels with inner retinal thinning in AD and other neuroinflammatory conditions (29, 30). Although these data remain preliminary, they support the possibility that retinal structural change may reflect glial-driven neurodegenerative processes.

Higher plasma p-tau217 levels were also associated with thinner INL, IPL, and GCL measurements, particularly in parafoveal regions. This is consistent with prior evidence of retinal ganglion cell loss in AD (31), retinal ganglion cell vulnerability to pathogenic tau (32), and retinal p-tau217 accumulation in the INL, IPL, and OPL of postmortem AD eyes (33). Together, these findings support the biological plausibility of a retinal tau-related signal in cognitively normal adults at increased risk for AD.

By contrast, associations with p-tau181 were weaker, and the NfL results should be interpreted only as preliminary trends because the confidence intervals were wide and the bootstrap-based *p*-values were not significant. Because an abnormal Aβ42/40 ratio of <0.10 was used as an inclusion/enrichment criterion, associations involving the Aβ42/40 ratio as an outcome may be affected by restricted range and selection bias and should be interpreted as exploratory only.

Using our novel AI retinal segmentation algorithm, our results more accurately resolved discrete inner retinal structures, such as the RNFL, GCL, IPL, and INL, that may be differentially affected across disease stages. Studies evaluating the relationship between plasma AD biomarkers and retinal layer thicknesses during the pre-clinical AD stage remain limited at this time. Ravichandran et al. (34) studied 82 cognitively unimpaired adults and found a significant association between plasma p-tau217 and retinal gliosis, while a multimodal model combining inner-sector RNFL thickness with plasma p-tau217 and Aβ42/40 discriminated preclinical AD. The RNFL putative gliosis, based on an analysis of only the inner retinal layers, likely reflected the inflammatory effects of the disease (11, 12). We did not confirm this finding in this study. In our previous pilot study of 11 cognitively healthy older adults classified as preclinical AD by a plasma Aβ42/40 ratio <0.10, (35) we used deep-learning SD-OCT segmentation to quantify ALL retinal layers: RNFL, GCL, IPL, and other inner, outer, and choroidal layers across ETDRS sectors. Photoreceptor inner segment thinning showed the largest association with plasma neurofilament light chain, while additional thinning or thickening across the other measured retinal layers was associated with higher plasma GFAP, p-tau181, and p-tau217. Amongst all of the retinal layers, the photoreceptor-inner segment layer thinning represents mitochondrial stress related to hypoperfusion from choriocapillaris flow deficits in these preclinical AD individuals with mostly ApoE-e4-negative alleles.

Overall, these findings suggest that retinal changes linked to blood-based AD pathology in the preclinical stage may be heterogeneous and layer-specific rather than confined to a single inner retinal measure. Notably, we hypothesize that the retinal layer thickness pattern may also depend on the ApoE-e4 status of the cohort, in which ApoE-e4 negativity would contribute to less inflammatory-related retinal layer thickness changes in relation to plasma biomarkers. Larger longitudinal studies comparing retina layer thickness patterns with plasma AD biomarkers in cohorts enriched with ApoE-e4-positive alleles vs. those with ApoE-e4 negative alleles will be required to confirm this hypothesis.

This study was not without limitations. Because this study was cross-sectional, retinal structure was measured at a single time point. We could not determine the temporal order in which different macular sectors changed relative to plasma biomarker trajectories. Because cohort enrichment was based on an abnormal plasma Aβ42/40, participants should be regarded as cognitively normal adults at increased risk for AD rather than definitively classified as having preclinical AD pathology. Because our study intentionally included only individuals with abnormal plasma biomarkers consistent with preclinical AD, we could not evaluate retinal layer thickness against normative aging patterns. Our results aimed to show the associations observed within abnormal cases and were not intended to compare normal and abnormal populations directly. The analysis therefore emphasized continuous relationships within the preclinical AD range. Future studies will include age-matched, biomarker-negative controls to establish normative benchmarks to enhance interpretation. Furthermore, participants were operationally classified as preclinical AD based on biomarker enrichment rather than definitive amyloid-confirmed diagnosis. Although amyloid PET scans were not performed in this study cohort, the CSF Aβ42/tau ratios have diagnostic performance comparable to amyloid PET for identifying AD-related amyloid pathology and early biologic AD. In a direct head-to-head BioFINDER/ADNI comparison (36), the best CSF measures were Aβ42/t-tau and Aβ42/p-tau with AUCs 0.93–0.94, while the best PET measures were 0.92–0.93. The authors concluded there were no differences between the best CSF and PET measures, and that the choice could be based on availability, cost, and patient/physician preference. CSF t-tau/Aβ42 and p-tau/Aβ42 ratios showed 89–90% overall agreement with amyloid PET and AUCs of 94–96%; these ratios were as accurate as semiquantitative PET image assessment for predicting visual-read outcomes (37).

Despite these limitations, our study has several important strengths, including detailed neurological and neuropsychological characterization of participants, exclusion of major ocular confounders, and use of a deep-learning-based segmentation pipeline with manual review of every B-scan. If validated, OCT-derived retinal layer metrics could serve as scalable, non-invasive biomarkers to complement blood-based assays for early AD risk stratification.

## 5 Conclusion

Although plasma biomarkers provide molecular information, SD-OCT can measure microscopic structural changes in the eye that may appear before any structural brain changes can be seen on conventional neuroimaging techniques, such as MRI and PET scans. These noninvasive retinal imaging techniques are also performed faster and are less expensive, with less radiation exposure than a PET scan. In a multimodal framework, retinal layer thickness could offer complementary information about spatial–temporal patterns of neurodegenerative change. In this proof-of-concept pilot study, layer- and region-specific inner retinal changes showed preliminary associations with plasma GFAP and p-tau217 in cognitively normal adults with an increased risk for AD. These results serve as the foundation for the design and powering of future larger longitudinal studies.

## Data Availability

The original contributions presented in the study are included in the article/supplementary material, further inquiries can be directed to the corresponding author.
